# Biodiversity and Ecosystem Functioning in Naturally and Experimentally Assembled Communities

**DOI:** 10.3390/biology12060835

**Published:** 2023-06-09

**Authors:** Daniel Puppe, Panayiotis G. Dimitrakopoulos, Baorong Lu

**Affiliations:** 1 Leibniz Centre for Agricultural Landscape Research (ZALF), 15374 Müncheberg, Germany; 2 Department of Environment, University of the Aegean, 81132 Mytilene, Greece; 3 Department of Ecology and Evolutionary Biology, School of Life Sciences, Fudan University, Shanghai 200333, China

Numerous studies have proved that biodiversity and ecosystem functioning (BEF) are closely linked. In this context, interactions between biodiversity and ecosystem functions, such as biomass production, trophic transfer through plants and animals, and pollination, have been in the focus of research [1,2,3]. Based on this, we now know that biodiversity promotes biomass production and pollination success, for example. However, regarding other BEF relationships, the drawing of general conclusions is hampered by the fact that different studies often show inconsistent results. Thus, for a better understanding of BEF relationships, detailed research on the underlying mechanisms is urgently needed. This knowledge is crucial for harmonizing research findings and to craft policies for the conservation of biodiversity, as it is severely threatened by global warming and other anthropogenic environmental changes (e.g., pollution, resource depletion, or species invasions).

In this Special Issue, two review and thirteen research articles dealing with a broad range of BEF relationships are published. The first review article provides a comprehensive overview of the current knowledge of autotroph–herbivore interactions in marine ecosystems, using macroalgae as a role model to describe the effects of macroalgal properties (e.g., nutrient composition or defense mechanisms) on herbivore feeding behavior [4]. The second review article presents METAL (MacroEcological Theory on the Arrangement of Life), which uses niche-environment interactions to explain phenomena such as phenology, biogeographical shifts, and community arrangement/reorganization [5]. The research articles in this Special Issue comprise studies on various forms of life (from microbiota, such as bacteria and fungi, to macrobiota, including tulips, monkeys, or giant pandas) in different terrestrial and aquatic ecosystems (e.g., grasslands, rivers, or marine hydrothermal vents), as well as in farming systems. In the first research article, wheat–faba-bean mixtures were studied to shed light on the phenomenon whereby increased diversity (e.g., represented by cereal–legume mixtures) in crop systems is often associated with higher yields and ecological sustainability compared to monocultures [6]. Let et al. [7] analyzed the effects of environmental pollution on the biodiversity (measured by abundances) of the larvae of aquatic insects, i.e., mayflies (Ephemeroptera), stoneflies (Plecoptera), and caddisflies (Trichoptera), in a river in central Bohemia (Czech Republic). The third research article deals with plant diversity and its relationships with soil microbes, i.e., bacteria and fungi, in semi-arid grassland in China, highlighting the critical role of soil microbe diversity in regulating soil multifunctionality [8]. Arunrat et al. [9] compared the microbial diversity, community composition, and functional structure of bacterial communities between rice–fish co-cultures and rice monoculture farming systems in Thailand to identify environmental driving factors of bacterial compositions in these systems. Man et al. [10] investigated microbes in *Sphagnum*-dominated peatlands in central China to unravel the linkages between different types of *Sphagnum* and the diversity and ecological functions of *Sphagnum*-associated microbiomes. In the sixth research article, the effects of village development on the habitat quality of the Yunnan snub-nosed monkey (*Rhinopithecus bieti*) in south China were evaluated to derive strategies for the protection of the habitats of these endangered monkeys [11]. Smeti et al. [12] performed a field study across nine rivers in Greece to analyze biodiversity–biomass relationships in benthic diatoms, which have been largely under-studied until now. Jia et al. [13] analyzed the distribution and microhabitat selection of the giant panda (*Ailuropoda melanoleuca*) in China to develop practical habitat conservation and management measures. In the ninth research article, rice straw and stubble burning in paddy fields in central Thailand was examined to assess changes in soil bacterial communities and soil properties after burning [14]. In the tenth research article, the plasticity of the grass *Aeluropus lagopoides* in saline habitats of Saudi Arabia was studied to better understand the interactions between the morphological parameters of plants and their environment [15]. Bilias et al. [16] analyzed the natural nutrient status and rhizosphere fungal morphotypes of wild-growing tulips in Greece to gain deeper insights into the adaptation of tulips to the environment. Cai et al. [17] examined crop-weed introgression (i.e., the transfer of genetic material from one species into the gene pool of another species) promoted by the change in rice cultivars to better understand weedy plant evolution and human influences in agroecosystems. Last but not least, Gheibzadeh et al. [18] studied alpha, beta, and gamma carbonic anhydrases (i.e., metalloenzymes that catalyze the hydration of carbon dioxide) in the thermophilic microbiome of marine hydrothermal vents to detect horizontal gene transfer, which is discussed as an important tool in maintaining microbial biodiversity in these harsh ecosystems.

All the articles in this Special Issue provide in-depth discussions on various aspects of BEF relationships in naturally and experimentally assembled communities. The articles in this Special Issue will help to substantially deepen our understanding of BEF interactions and to elaborate biodiversity conservation in a changing world. Therefore, the collection of these articles is of great interest not only for scientists in this field, but also for students, university teachers, and policymakers, as well as those who are interested in BEF in general.
